# Comprehensive analysis of the cerebellar vasculature of dromedary camels (*Camelus dromedarius*)

**DOI:** 10.3389/fvets.2024.1447881

**Published:** 2025-01-14

**Authors:** Ahmad Al Aiyan, Rinsha Balan

**Affiliations:** Department of Veterinary Medicine, College of Agriculture and Veterinary Medicine, United Arab Emirates University, Al Ain, United Arab Emirates

**Keywords:** dromedary camel, cerebellum, rostral cerebellar artery, caudal cerebellar artery, casting techniques

## Abstract

The current study performed a comprehensive assessment of blood supply in the cerebellum of dromedary camels. To the best of our knowledge, this study is the first to provide detailed information about the origins, routes, and complicated patterns of branching in the rostral and caudal cerebellar arteries of dromedary camels. In total, 55 heads from male dromedary camels aged 2–6 years were analyzed using advanced casting techniques. Based on the specific challenges of this study, arterial structures were accurately evaluated using these strategic techniques. Rostral and caudal cerebellar arteries and branches of the basilar artery gave rise to multiple cortical branches categorized as hemispheric (lateral, middle, and medial) and vermian (paramedian and median) branches, which supply blood to the cerebellum. This novel anatomical knowledge can significantly improve our understanding about the neurovascular system of dromedary camels, thereby holding potential implications for veterinary diagnostics, treatment of neurological disorders, and comparative neuroanatomy research.

## 1 Introduction

**Figure 1:**
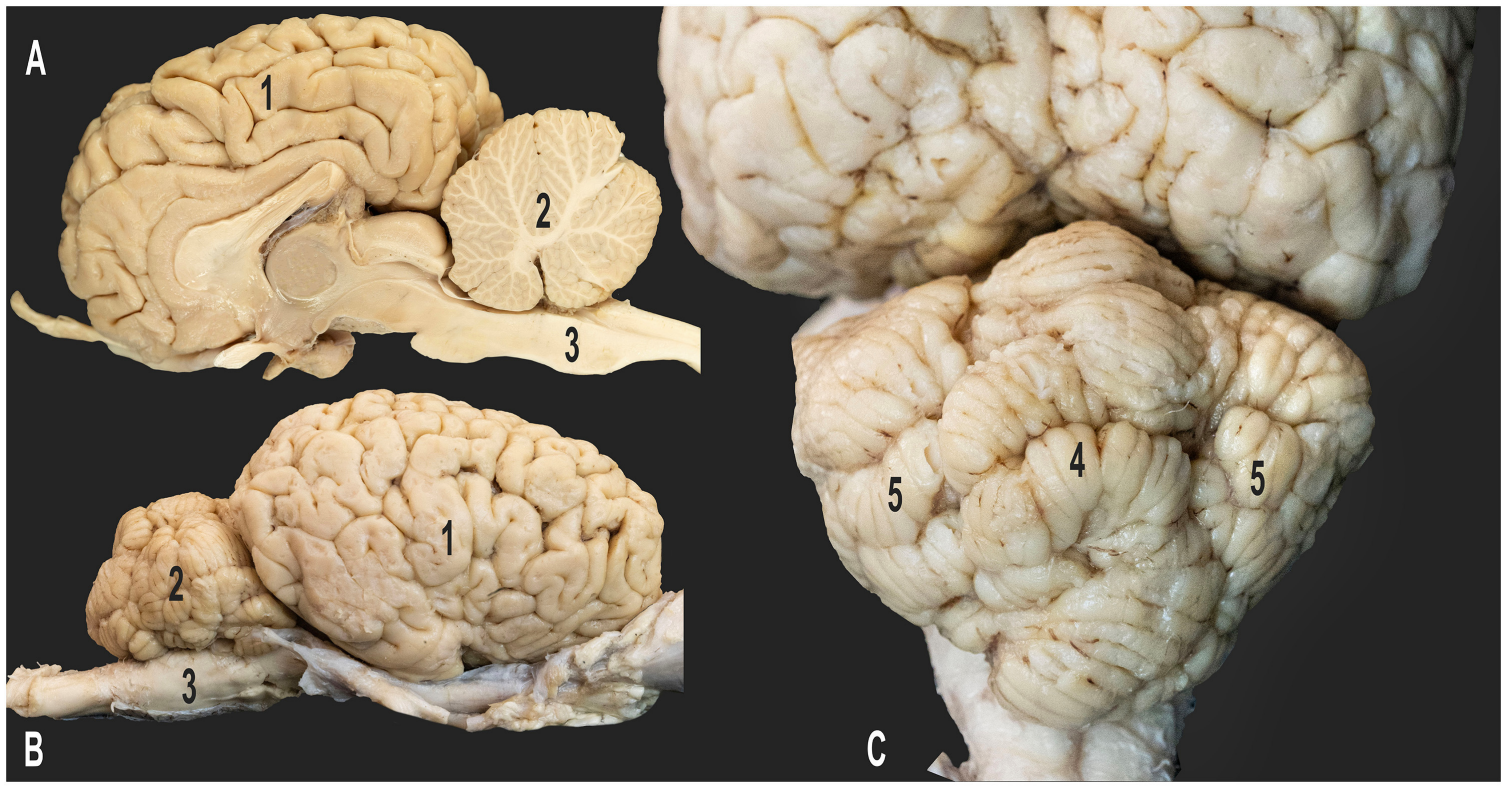
Gross anatomy of the dromedary brain. **(A)** Medial view of the right cerebral hemisphere; **(B)** lateral view of the right cerebral hemisphere; **(C)** caudo-dorsal view of the cerebellum; 1, right cerebral hemisphere; 2, cerebellum; 3, medulla oblongata; 4, vermis of the cerebellum; 5, right and left cerebellar hemispheres.

Hindbrain is one of the three major regions of the brain and is located at the lower back part of the brain. It includes most of the brainstem and a dense coral-shaped structure referred to as the cerebellum. The supply of blood to the cerebellum, also referred to as the little brain, has been an interesting research topic for centuries. The cerebellum is located caudal to the cerebral hemispheres, separated ventrally from the pons and medulla oblongata by the fourth ventricle, and cranially from the cerebrum by the tentorium cerebelli (Figure 1). The cerebellum comprises two symmetrical lateral halves, two cerebellar hemispheres that flank the medial part, and the vermis (1, 2). The dorsal surface of the camel's cerebellum has several fissures of various depths, subdividing the whole cerebellar surface into several leaf-like lamellae, which are separated by sulci of different depths (3). The cerebellum plays a fundamental role in diverse motor functions, postural control, balance, and motor coordination (4, 5). Further, it is involved in cognitive functions such as language, learning, memory, and some emotional behaviors, including fear (6, 7). In domesticated mammals, the brainstem and cerebellum are supplied with arterial blood from two distinct systems, the carotid and vertebrobasilar systems, which are connected via the circle of Willis. The vertebrobasilar arterial system supplies approximately 30% of the afferent arterial blood to the brain in dromedary camels (*Camelus dromedarius*). However, because of its route, it primarily supplies the cerebellum and is the only source of arterial blood supply to the medulla oblongata and pons (8, 9). The cerebellum receives its vascular supply mainly from the rostral and caudal cerebellar arteries, which are the two branches of the basilar artery.

The cerebellar artery is of great clinical importance and is associated with several diseases such as aneurysm, ischemic stroke, neurovascular compression syndrome, arteriovenous malformation, and brain tumors. These can result in impaired motor coordination, balance issues, and other neurological deficits (10, 11). A comprehensive understanding of the anatomy of the cerebellar arteries is required for prompt diagnosis and treatment to prevent further neurological damage and improve the animal's quality of life.

Despite the acknowledged importance of the cerebellar arteries, detailed descriptions of their branching patterns, particularly in animals, remain limited. While some anatomical studies have described the origin of the cerebellar arteries, they often overlooked a detailed examination of their branching patterns and supply routes. To the best of our knowledge, this study is the first to perform a comprehensive assessment of the rostral and caudal cerebellar arteries in dromedary camels. This detailed description could be of considerable value to both researchers and experts in the field.

This study aimed to comprehensively analyze the rostral and caudal cerebellar arteries of dromedary camels by examining their origins, courses, arrangements, branches, and anastomoses with other cerebral arteries. Advanced casting techniques were used to provide a precise three-dimensional description and elucidate the finer arterial branches of the cerebellar arteries in dromedary camels.

## 2 Materials and methods

This study was conducted in accordance with the ethical guidelines established by the United Arab Emirates University. Our study utilized heads with 3–7 cervical vertebrae from male Omani dromedary camels (*Camelus dromedarius*) aged 2–6 years (*n* = 55). These specimens were obtained from Al Ain City Municipality Camel Slaughterhouses.

The common carotid arteries of all samples were cannulated for casting agent injection according to the method used in our previous studies (12, 13). In total, 30 heads were injected with epoxy resin (Gulf Guard Epoxy), 20 with polyurethane resin (EasyFlo 60 Liquid Plastic), and 5 with latex neoprene (Latex Globalsil AL 20). The choice of various casting materials for the injections was a calculated move considering the unique challenges of our experimental research. Liquid polyurethane resin was chosen due to its ability to produce a solid but slightly flexible cast. This flexibility permits slight movements of the branches, which reduces the chance of breakage and helps maintain the intricate artery network. Conversely, epoxy resin was used to form a very rigid cast. This characteristic was perfect for achieving a precise 3D representation of the arteries in our study. However, this type of resin's lack of flexibility made the arteries and branches more exposed to breaking. Additionally, to extract the brains while protecting the arteries, we also made use of a red latex solution. This material provides high flexibility, which is beneficial for this specific stage of the procedure. The injection volume, ranging 400–800 mL, was determined based on head size and the number of cervical vertebrae present. Manual injection was performed using 60-mL syringes. To solidify the casting agents, the injected heads were refrigerated at 5°C for a minimum of 24 h.

After ensuring that the casting materials had stiffened, the cranial roof and vertebrae were opened using a rotating power saw (DeWalt DWE4001 with a 16-mm blade). Three-dimensional representations of the cerebral vasculature were created using various techniques. In 20 specimens, high-pressure water was used to remove brain tissues while preserving the epoxy/polyurethane casts. In 10 specimens, 5% potassium hydroxide solution was utilized to remove non-cast tissues, including soft tissues and bones. In another group, sodium carbonate solution with a temperature of 60°C was used to digest soft tissues, leaving the bone and cerebral arteries intact. Finally, the tissues of five latex-injected heads were fixed in 6% formaldehyde for 2 weeks. Next, cautious dissection was conducted to extract the brain.

## 3 Results

**Figure 2:**
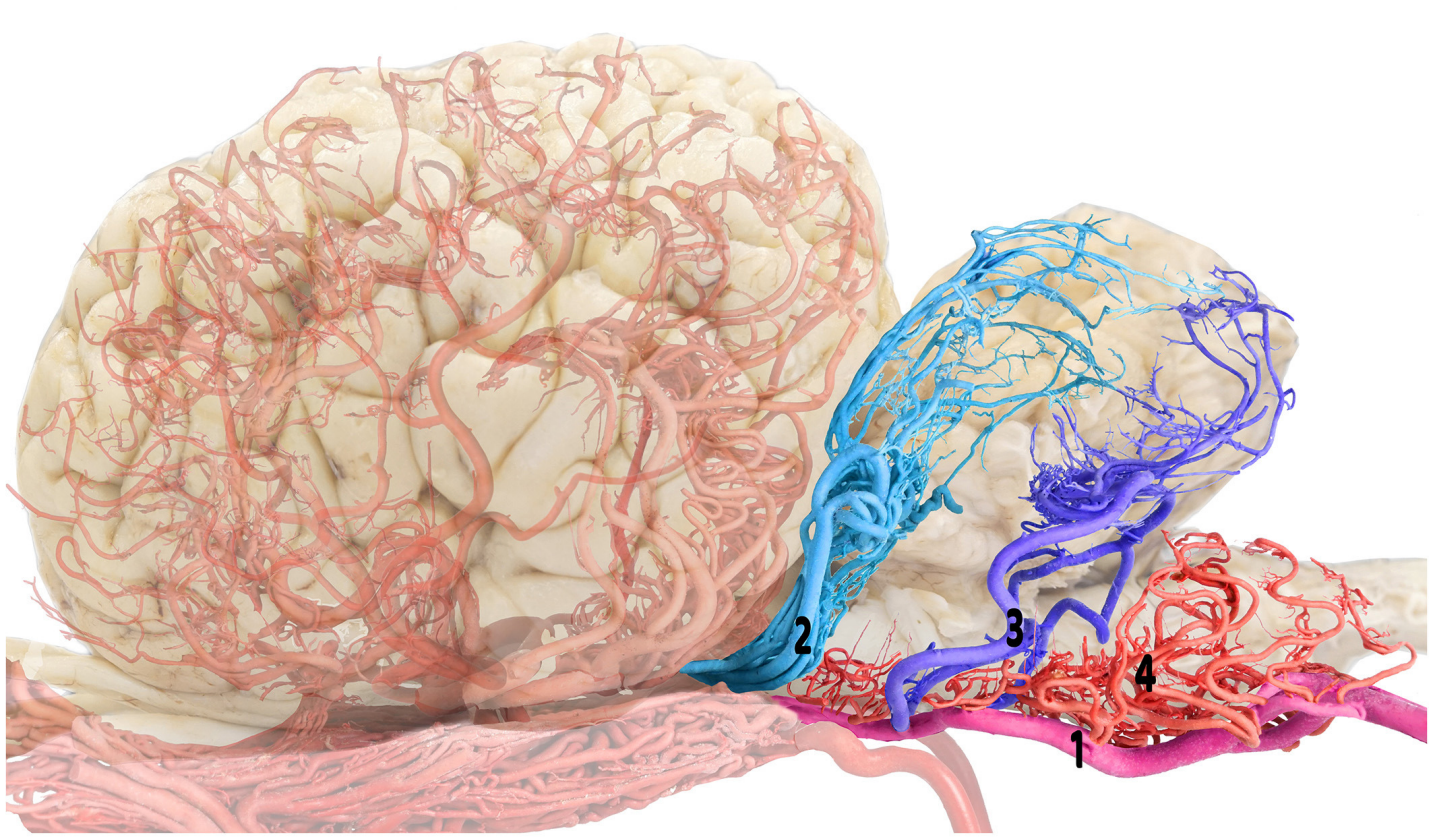
Composite visualization of the camel cerebellar arterial supply emphasizing the rostral and caudal cerebellar arteries: brain shadow overlay. 1, basilar artery; 2, rostral cerebellar artery; 3, caudal cerebellar artery; and 4, medullary branches.

**Figure 3:**
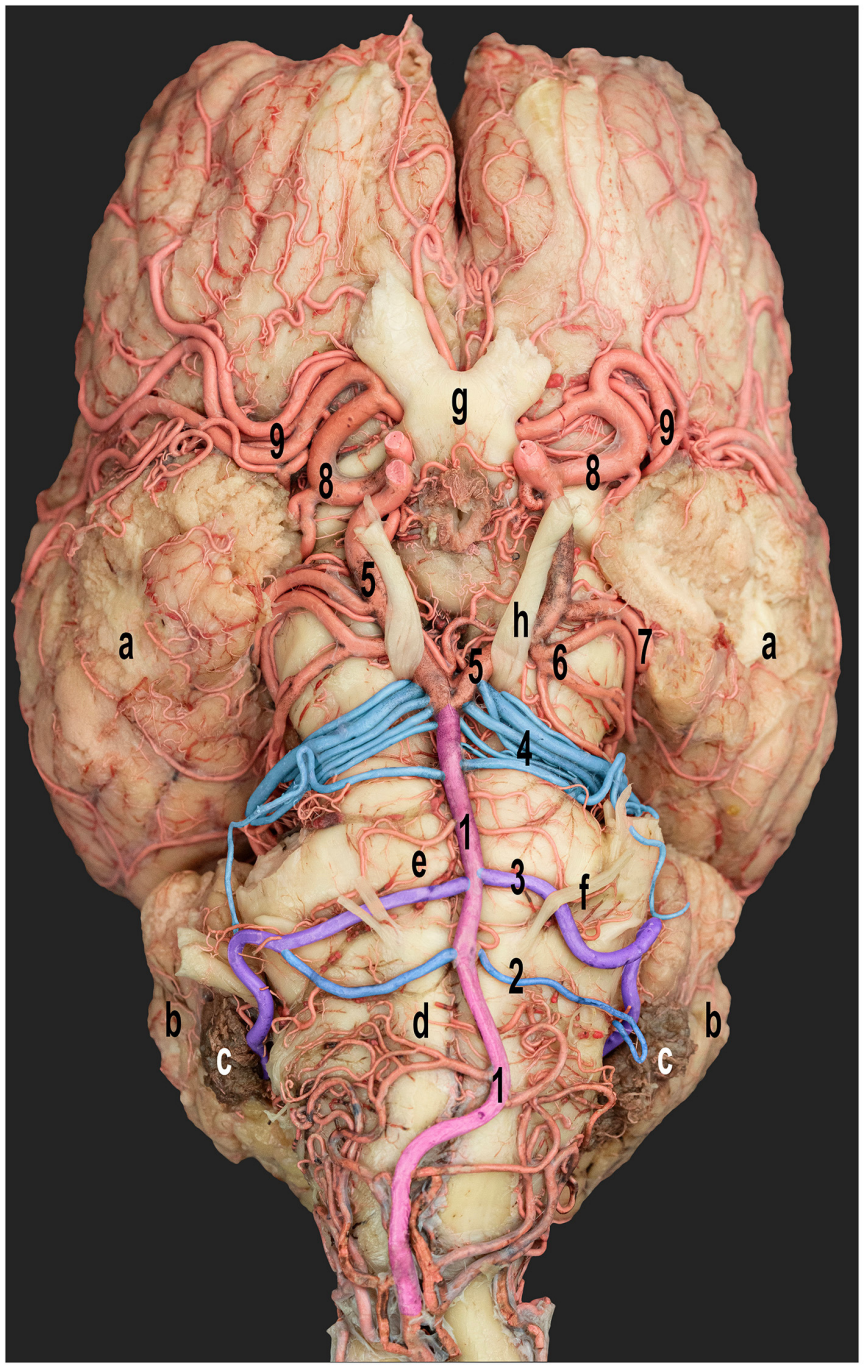
Ventral view of the dromedary camel brain emphasizing the cerebellar arterial supply. 1, basilar artery; 2 and 3, rostral and caudal roots of the caudal cerebellar artery; 4, rostral cerebellar artery; 5, caudal communicating artery; 6, caudal cerebral artery; 7, caudal choroidal artery; 8, rostral cerebral artery; and 9, middle cerebral artery; a, cerebral hemispheres; b, cerebellar hemispheres; c, choroid plexus of the fourth ventricle; d, medulla oblongata; e, pons; f, abducent nerve; g, optic chiasm; and h, oculomotor nerve. Right side-rostral and caudal roots of the caudal cerebellar arteries fused to form a single trunk.

**Figure 4:**
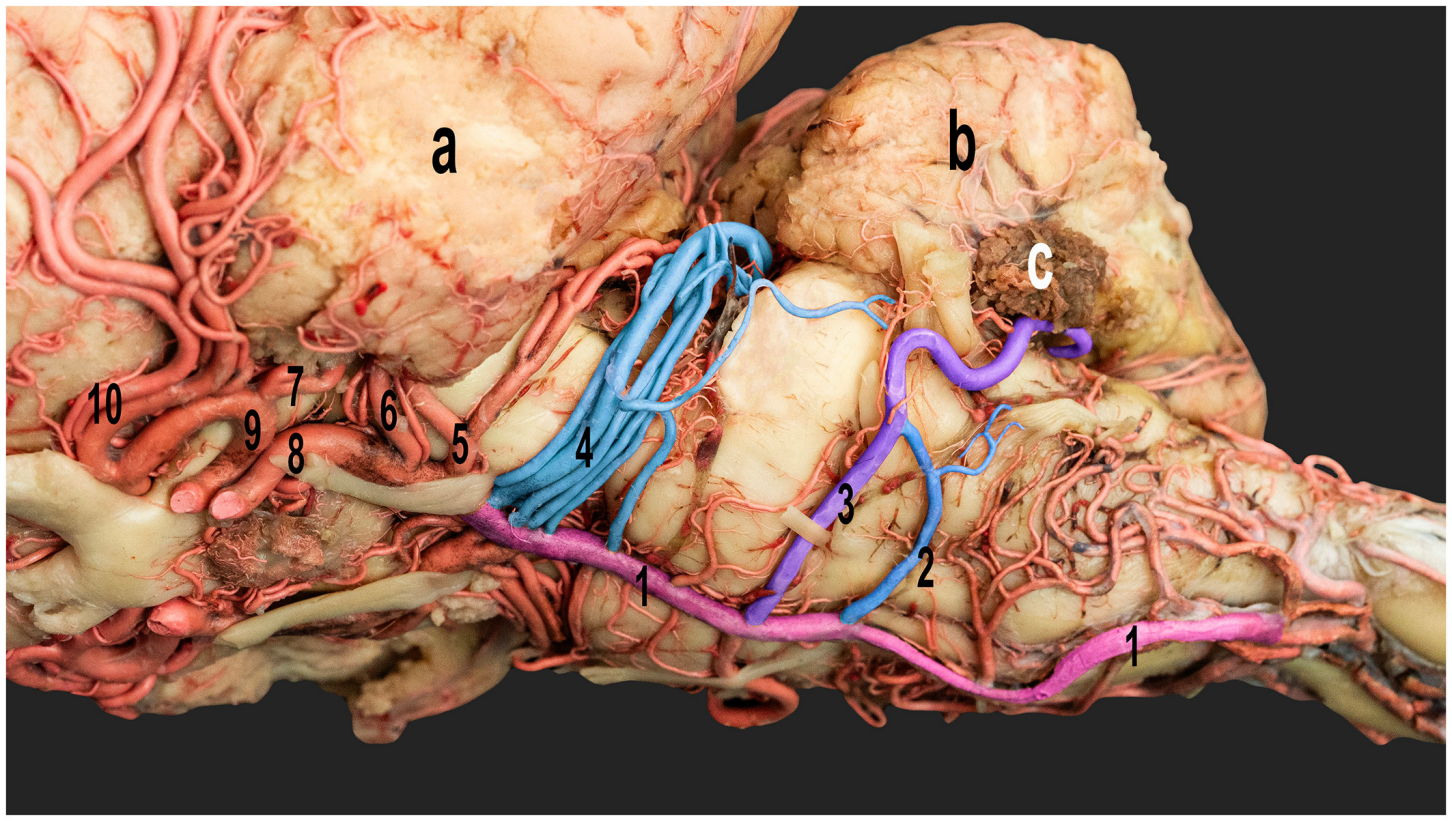
Ventrolateral view of the dromedary camel brain emphasizing the cerebellar arteries originating from the basilar artery. 1, basilar artery; 2, caudal root of the caudal cerebellar artery; 3, rostral root of the caudal cerebellar artery; 4, rostral cerebellar artery; 5, caudal cerebral artery; 6, caudal choroidal artery; 7, rostral choroidal artery; 8, caudal communicating artery; 9, rostral cerebral artery; and 10, middle cerebral artery; a, left cerebral hemisphere; b, cerebellum; c, choroid plexus of the fourth ventricle.

This study examined arterial supply to the cerebellum in camels. Arterial supply to the cerebrum, cerebellum, and brainstem was provided by the carotid and vertebrobasilar arterial systems, which are two distinct systems. The vertebrobasilar system comprises the basilar artery, which is formed by the fusion of the two medial branches of the vertebral arteries with the ventral spinal arteries. Meanwhile, the vertebrobasilar system includes the lateral branches of the vertebral arteries, which are connected to the branches of the basilar artery that supply oxygenated blood to the medulla oblongata. In camels, the basilar artery exhibits a unique characteristic. It is supplied exclusively to the cerebellum via the rostral and caudal cerebellar arteries (Figures 2–4).

Based on our research findings, the basilar artery followed the course along the ventral surface of the medulla oblongata within the ventral median fissure after its formation. Notably, the basilar artery gave off distinctly thin, tortuous medullary branches, which resembled a mesh-like structure supplying the ventral surface of the medulla oblongata (Figures 3, 4). These branches were laterally distributed and extended to the dorsal aspects of the medulla oblongata. Moreover, the medullary branches presented with extensive anastomoses. The medullary branches were anastomosed with the fine branches of the caudal cerebellar artery, which arose more rostrally from the basilar artery (Figures 3, 4). In addition, anastomoses were observed with the terminal branches of the vertebral artery's lateral branch at the level of the caudal cranial fossa.

As the basilar artery coursed rostrally, it gave rise to the rostral and caudal roots of the caudal cerebellar arteries, which were two prominent pairs of arteries at the rostral end of the medulla oblongata and the caudal border of the pons (Figures 3, 4). In the examined samples, the rostral and caudal roots of the caudal cerebellar artery emerged from the lateral surface of the basilar artery. Moreover, they coursed laterally at the lateral aspect of the medulla oblongata before coursing caudolaterally, crossing over the abducens nerve. Interestingly, in a small subset of the samples, these roots fused to form the main trunk of the caudal cerebellar artery (Figures 3, 4).

**Figure 5:**
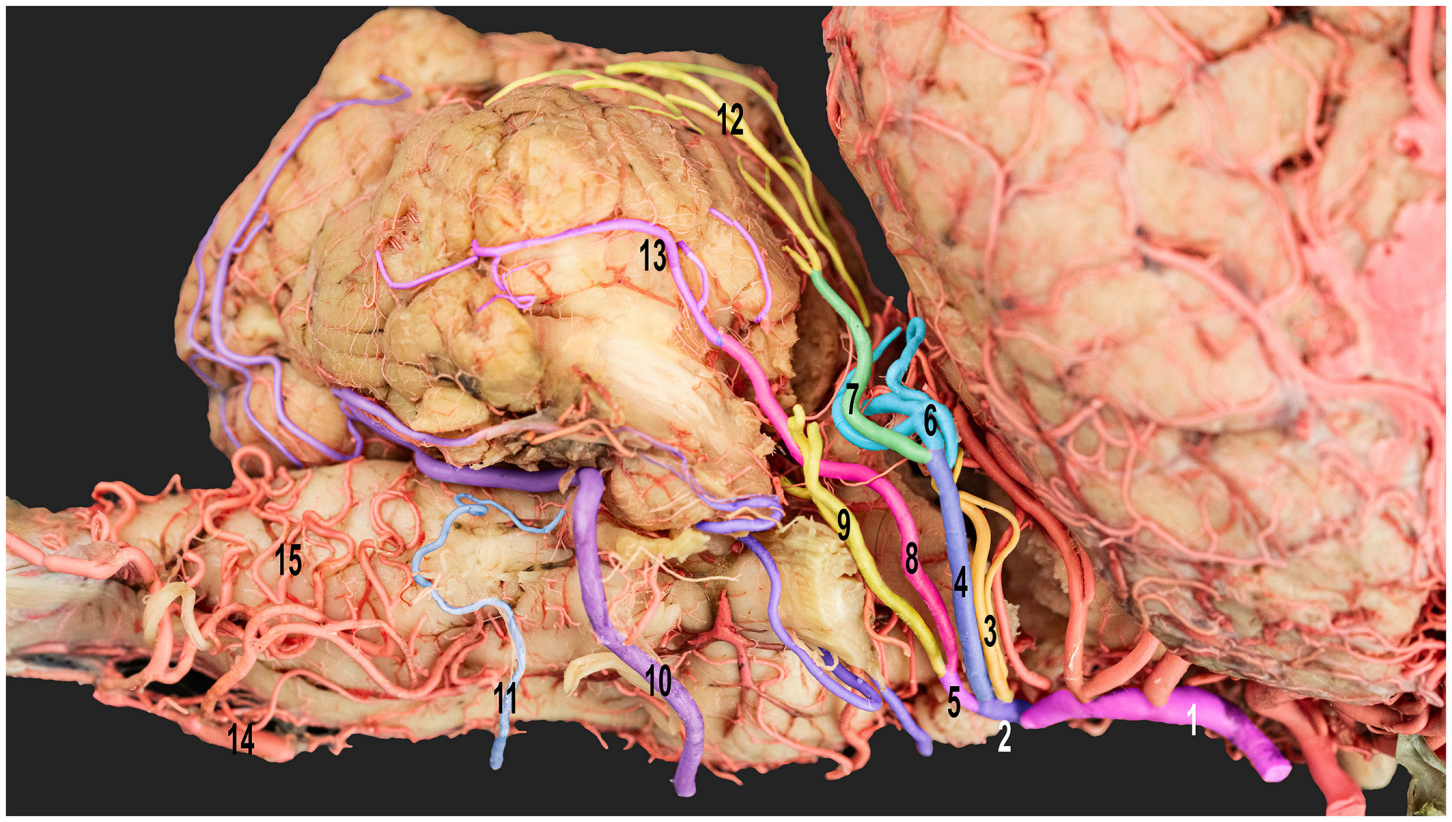
Ventrolateral view of the right half of the dromedary camel's brain emphasizing the cortical branches of the right cerebellar artery. 1, caudal communicating artery; 2, rostral cerebellar artery; 3, precerebellar artery; 4, medial branch of the rostral cerebellar artery; 5, lateral branch of the rostral cerebellar artery; 6, medial branch of the medial rostral cerebellar artery; 7, lateral branch of the medial rostral cerebellar artery; 8, medial branch of the lateral rostral cerebellar artery; 9, lateral branch of the lateral rostral cerebellar artery; 10, rostral root of the caudal cerebellar artery; 11, caudal root of the caudal cerebellar artery; 12, vermian branches; 13, hemispheric branches; 14, basilar artery; and 15, medullary branches.

**Figure 6:**
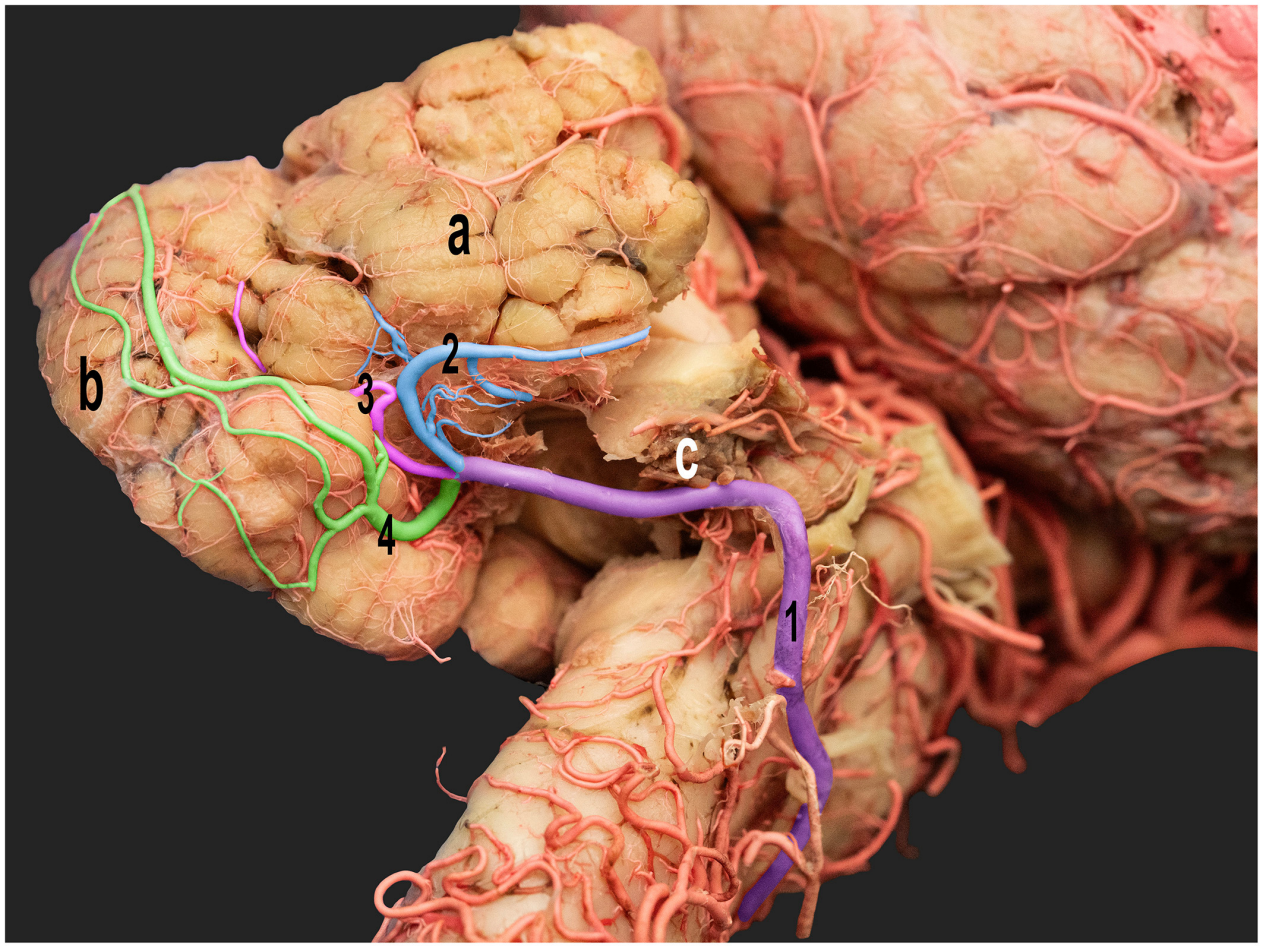
Ventrolateral view of the right half of the dromedary camel brain highlighting the cortical branches of the right caudal cerebellar artery. 1, caudal cerebellar artery; 2, lateral hemispheric branch; 3, middle hemispheric branch; 4, medial hemispheric branch; a, right cerebellar hemisphere; b, vermis; c, choroid plexus.

The caudal cerebellar artery ascended from a laterodorsal trajectory, giving rise to numerous cortical branches directly from the main trunk. These cortical branches can be classified into two distinct groups: hemispheric and vermian branches. As the caudal cerebellar artery ascends, it sequentially gives rise to the lateral, middle, and medial hemispheric branches, followed by the paramedian and median vermian branches. Thereafter, the caudal cerebellar artery crosses to the contralateral side, passing caudoventrally to the cerebellum, where it forms an anastomosis with the corresponding caudal cerebellar artery from the opposite side (Figures 5, 6).

The lateral hemispheric branch ran caudally to the facial nerve, supplying the lateral surface of the cerebellar hemisphere. The middle and medial hemispheric branches were larger. Moreover, they followed the groove between the vermis and the lateral cerebellar hemisphere, ascending medially in a curved trajectory. They formed dorsal arches over the caudal cerebellar surface, supplying blood to the region (Figures 5, 6).

The vermian branches arose from the caudal cerebellar artery within the vermohemispheric fissure, supplying the median and paramedian vermian areas (Figure 6).

The trajectories of several small branches originating from the main trunk of the caudal cerebellar artery coursed alongside the facial and vestibulocochlear nerves, ultimately reaching the dorsal surface of the brainstem. These arteries sent out branches to supply the middle and caudal cerebellar peduncles while vascularizing the choroid plexus of the fourth ventricle and the superolateral aspect of the medulla oblongata. These arteries were characterized by extensive branching and numerous anastomoses with the medullary branches, forming a complex vascular network on the medullary surface (Figures 3–5). The configuration of these anastomoses demonstrated some variability across the studied specimens, particularly at the level of the terminal small branches.

The caudal cerebellar artery gave rise to the choroidal arteries that supply the choroid plexus of the fourth ventricle. The labyrinthine artery is a small branch from the rostral root of the caudal cerebellar artery that moves laterally into the internal auditory meatus along with the vestibulocochlear nerve.

After giving rise to the prominent caudal cerebellar arteries, the basilar artery coursed through the basilar groove located on the ventral aspect of the pons. The pontine arteries arose from the basilar artery at right angles. Further, they were characterized by their thinness, short length, even spacing, and parallel orientation. Based on our findings, some arteries also anastomosed with the early branches of the caudal cerebellar arteries.

At the level of the interpeduncular fossa, the basilar artery anastomosed with the two caudal communicating arteries arising from the rostral epidural rete mirabile (RERM) to form the caudolateral wall of the cerebral arterial circle (Figure 3). Our study observed that the rostral cerebellar artery was formed by a group of 4–6 branches, with notable variations in the number and configuration of these branches across the studied specimens (Figures 3, 4). In 20% of the studied cases, the basilar artery released 3–4 branches before anastomosing with the caudal communicating artery. However, in 60% of the specimens, only two branches arose directly from the basilar artery, while additional branches originated from the junction between the caudal communicating and basilar arteries. In 20% of the studied samples, the first two branches were observed to arise from the caudal communicating artery before anastomosing with the basilar artery. These findings underscore subtle inter-individual differences in the arterial network contributing to the formation of the rostral cerebellar artery.

**Figure 7:**
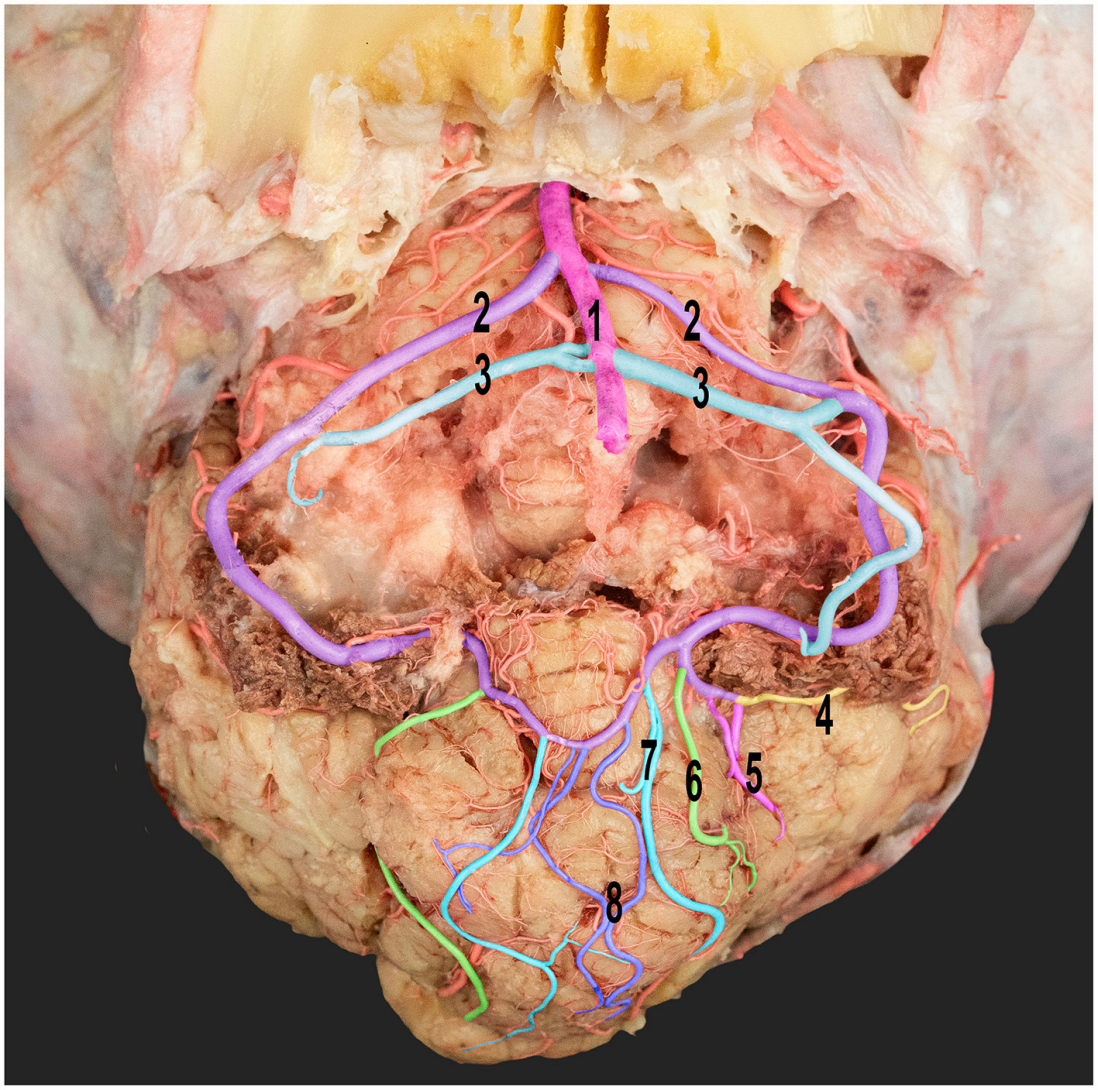
Ventral view of the dromedary camel's cerebellum with the medulla oblongata removed, thereby revealing the cortical branching pattern of the caudal cerebellar artery. 1, basilar artery; 2, rostral root of the caudal cerebellar artery; 3, caudal roots of the caudal cerebellar artery; 4, lateral hemispheric branch; 5, middle hemispheric branches; 6, medial hemispheric branch; 7, paramedian vermian branch; and 8, median vermian branch.

According to our research, the pre-cerebellar arteries were the first two branches arising from the caudal communicating artery (Figure 7). These arteries play an important role in supplying blood to the rostral and middle cerebellar peduncles and the caudal and rostral colliculi. The remaining branches of the rostral cerebellar artery traveled dorsally along the curvature between the rostral and middle cerebellar peduncles. In addition, they provided blood supply to the rostral part of the cerebellum.

The rostral cerebellar artery ran above the trigeminal nerve and was divided into two branches, namely, the medial and lateral branches (Figure 7). The medial branch further bifurcated into two additional branches (Figure 7). These branches supply the rostral part of the vermis and adjacent surfaces of the cerebellar hemisphere. Moreover, they sent rami to the dental nucleus and contributed to other deeper nuclei in the cerebellar hemisphere. The lateral branch of the cerebellar arteries is divided into medial and lateral branches (Figure 7), which supply blood to the deep and superficial lateral surfaces of the cerebellar hemispheres.

**Figure 8:**
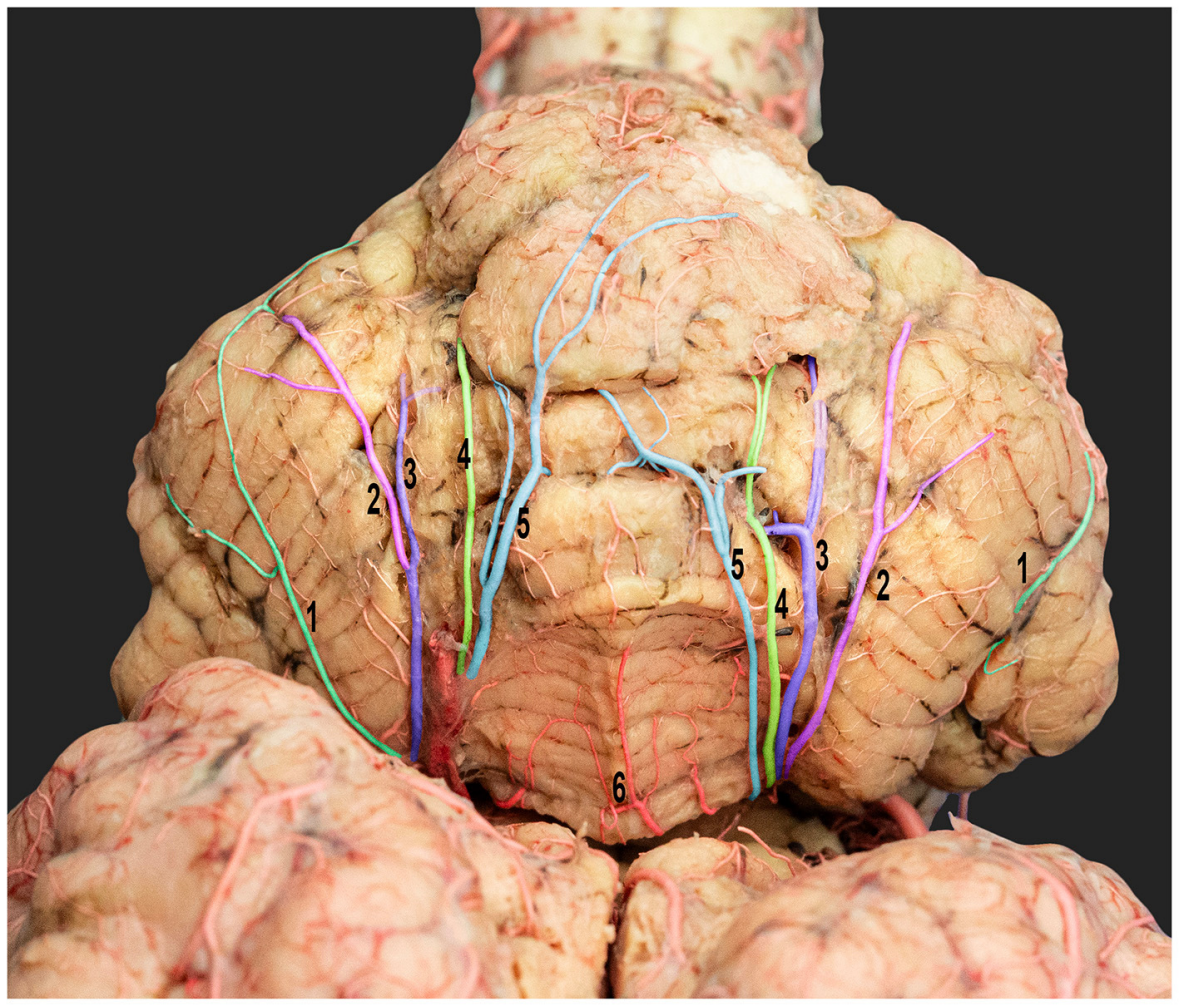
Dorsorostral view of the dromedary cerebellum emphasizing the standard cortical branching pattern of the rostral cerebellar artery. 1, lateral hemispheric branches; 2, intermediate hemispheric branches; 3, medial hemispheric branches; 4, paramedian vermian branches; 5, median vermian branches; and 6, vermis.

The rostral cerebellar artery traveled along a path toward the transverse fissure. It ran through the groove, separating the vermis and the lateral cerebellar hemisphere over the rostral surface of the cerebellum. It then sent out multiple cortical branches, including the hemispheric and vermian branches (Figures 7, 8), which supply the cerebellar cortex. The hemispheric branches arose from the rostral cerebellar artery's medial and lateral branches and from its main trunk. The hemispheric branches are divided into three types (lateral, intermediate, and medial) (Figure 8), each supplying the corresponding cerebellar hemispheric surface. These hemispheric branches divided into sub-branches, arborized over the tentorial surface, and disappeared between the cerebellar folia. The vermian branches arose from the medial and lateral branches of the rostral cerebellar artery. There were two types of vermian branches: the medial and paramedian branches (Figure 8). The medial branch supplied the middle portion of the rostral and dorsal aspects of the vermis. Meanwhile, the paramedian branch was distributed paramedially in the hemispherical zone. A frequent anastomosis was observed between the vermian branches from both sides near the apex of the tentorial surface. These branches provided the rostral portion of the sub-occipital surface and the upper two-thirds of the petrosal surface.

Small branches originated from the main trunk and from the medial and lateral branches of the rostral cerebellar artery. These branches coursed around the brain stem, the rostral and middle cerebellar peduncles, and the rostral and caudal colliculi of the mesencephalon.

While the overall branching pattern of the cerebellar arteries was consistent across the specimens, some variability was observed in the origins of the terminal branches of both the rostral and caudal cerebellar arteries across the examined specimens, specifically, the number and configuration of the smaller vessels arising from the main hemispheric and vermian branches.

## 4 Discussion

To the best of our knowledge, this study is the first comprehensive analysis of the origin, course, and branching pattern of the cerebellar arteries in dromedaries. While previous studies have identified the origin of the major cerebellar arteries in various animal species, detailed descriptions of their branching patterns, courses, and anastomoses are often lacking. It is important to validate that any comparisons made between our findings in camels and those in other animals refer to the origin of these arteries, not their branches. Several studies have shown that cerebellar arteries contribute to the blood supply to the brain in various animal species such as donkey (equine), deer, cattle (bovine), goat (caprine) and elk (cervid) (14–18). However, the intricate details of their branching pattern and course have not been discussed. Furthermore, these studies did not present a comprehensive description or consider the significant variations observed in camel vasculature (1, 19–21). Due to a lack of previous research that accurately characterizes and identifies the branches of the cerebellar arteries in animals, this study aimed to gain a deeper understanding of the anatomy of cerebellar arteries and their branches in camels. In this study, we evaluated the branches and course of the cerebellar arteries based on the regions they supplied.

The vertebrobasilar arterial system is a supplementary afferent arterial system that contributes directly to the circle of Willis. The vertebral arteries add to the ventral spinal artery to form the basilar artery that anastomoses with the caudal communicating arteries. Moreover, they constitute the caudal wall of the cerebral arterial circle. This arterial system transports blood directly from the subclavian arteries, and it is pulsatile in nature (22, 23). Unlike the RERM, this channel is a direct connection from the heart and forms a significant supply to the brain.

Our study showed that the basilar artery supplies the cerebellum, medulla oblongata, and pons directly before joining the cerebral arterial circle. The basilar artery sequentially gives rise to the medullary, caudal cerebellar, pontine, and rostral cerebellar arteries, starting from the caudal aspect of the medulla. Furthermore, the rostral cerebellar artery originates from the basilar–caudal communicating anastomosis. The artery can have multiple points of origin and mainly stems from the rostral part of the basilar artery. In some cases, the first branches of the rostral cerebellar arteries originate from the caudal communicating arteries. In contrast, in other samples, they originate from the basilar artery and caudal communicating artery junction and travel caudally to supply the cerebellum. A similar observation was reported in giraffes. That is, the rostral cerebellar arteries branched off the caudal communicating arteries near the junction of the caudal communicating arteries and the basilar artery. In some giraffes whose arteries branched out directly from the basilar artery, the most significant variation was observed in the rostral cerebellar arteries (24). Zdun et al. (18) reported that the rostral cerebellar arteries branch from the caudal communicating arteries in elks. In this study, some elks have rostral cerebellar arteries that branch out in two ramifications. One of these branches is a part of the caudal communicating artery. Meanwhile, the other is a part of the basilar artery (18). According to Zdun et al. (16), the rostral cerebellar artery in Bovidae comprises one or more arteries that branch from the caudal communicating artery at approximately half its length. In some Cervidae species, the rostral cerebellar artery is formed by multiple branches from the caudal communicating artery and the basilar artery (15). This artery has similar branching patterns in roe deer (25), camelids (20), and Bovidae (16).

Alsafy et al. (17) showed that the rostral cerebellar arteries descended from the caudal communicating artery in goats (caprine). This finding differs from that of the study of Kapoor et al. (26), which revealed that the rostral cerebellar artery was a branch of the caudal cerebral artery in goats. The rostral cerebellar artery in the yak (27) arises from a branch of the mesencephalic artery, crosses the cerebral crus, and ascends dorsally and caudally between the cerebellar hemisphere and caudal colliculus. Monkeys are the only animals whose rostral cerebellar artery arises exclusively from the basilar artery (26). It emerges from the caudal cerebral artery in dogs, rabbits, goats, and sheep (26).

According to Gillilan (28), the superior cerebellar artery originates from the basilar artery, encircles around the brainstem, and distributes to the cerebellum dorsally and rostrally in pigs. The superior cerebellar arteries of ox and sheep arise symmetrically at the caudal divisions of the internal carotid arteries just before they join to form the basilar artery, as these vessels extend around the brainstem and supply the cerebellum, vermis, and middle cerebellar peduncles (28).

Our study revealed that the rostral cerebellar artery is divided into two branches (the medial and the lateral branches) after crossing the trigeminal nerve. This structure is also observed in humans (29). Therefore, the superior cerebellar artery originates from the basilar artery (26, 29). Our study also found that the medial branch leads to two medial and lateral branches. Moreover, the rostral cerebellar artery supplies blood to various cortical branches, including the hemispheric and vermian branches. These branches supply the rostral dorsal aspect of the cerebellar hemispheres, vermis, and rostral and caudal colliculi of the mesencephalon. The rostral cerebellar arteries of dogs and cats supply the vermis, dorsolateral brainstem, and rostral portion of the cerebellar hemispheres (30, 31). In addition to these cortical branches, the rostral cerebellar artery sends several small branches to the brainstem and cerebellar peduncles. Because of the lack of information on animal brains, our findings regarding the branches of the cerebellar arteries could not be compared with any current animal studies.

Our study observed that the rostral cerebellar and caudal cerebral arteries move in different directions and supply different regions of the camel brain. In this study, the epoxy cast models of both arteries presented with an evident demarcation between the region of supply of both arteries. They do not seem to anastomose with each other, thereby contradicting previous observations that show communicating channels between the two arteries (20, 32). Khairuddin et al. (33) revealed differences between the caudal cerebral and rostral cerebellar arteries in donkeys.

In all evaluated samples, the caudal cerebellar artery showed two points of origin from the basilar artery at the caudal border of the pons. This artery had similar patterns of origin in a dromedary (19, 34), Bactrian camel (1), llama and guanaco (20), giraffe (24), Bovidae (16), and Eurasian elks (18). The caudal cerebellar artery in yak (27) originates from the basilar artery at the level of the junction of the trapezoid body and the pons rostral to the abducent nerve root. The inferior cerebellar artery of the pig is derived from the basilar artery at the lower border of the pons, where it crosses the abducens nerve root fibers and travels laterally to the area of the facial and auditory nerves and gets distributed to the cerebellum's inferior and caudal surfaces (28). Zdun et al. (16) revealed vascular variations in bovine fetuses and newborn calves. The right caudal cerebellar artery originated close to the junction between the basilar and the right caudal communicating artery. Then, it ran parallel to the basilar artery. After reaching the levels of the left caudal cerebellar artery and turning to the right, it directed itself toward the cerebellar hemisphere.

Kiełtyka-Kurc et al. (20) observed a double caudal cerebellar artery in Bactrian camel, llama, and guanaco. Several species such as Equidae (35, 36), Felidae (37), and giraffe (24) presented with double caudal cerebellar arteries. Kiełtyka-Kurc et al. (15) observed several caudal cerebellar and pontine arteries branching off the basilar artery in the family Cervidae. Zdun et al. (18) reported the same finding in yaks.

Our study revealed that the caudal cerebellar artery travels dorsolaterally to the caudal cerebellar peduncle and then ascends diagonally toward the back of the cerebellum, branching into numerous hemispheric (lateral, middle, and medial) and vermian (paramedian, median) arteries that supply the lateral and caudal aspects of the cerebellar cortex. After giving off these branches, the common carotid artery crosses to the opposite side and connects with the corresponding artery from the other side.

In humans, the inferior cerebellar artery divides into two branches: the rostrolateral and caudomedial branches. These branches divide after crossing the facial and vestibulocochlear nerves (29). Gillilan (28) showed that the inferior cerebellar arteries emerge symmetrically from the basilar artery at the level of the abducens nerves in ox. These arteries then divide into two branches at the end of the cerebellum. One of the branches spreads and connects with the superior cerebellar arteries, whereas the other branch descends posterolaterally along the brainstem to supply blood to the lateral and posterior funiculi.

The caudal cerebellar artery sends multiple cortical branches to the caudoventral cerebellum, including the hemispheric and vermian branches. These cortical branches supply various surfaces of the hemispheres, including the petrosal, tentorial, medial, and lateral surfaces, as well as the vermian area and sub-occipital surfaces. This finding was also observed in humans. That is, several cortical branches emerge from the inferior cerebellar artery and radiate outward to the medial and lateral hemispheres and the medial and paramedian vermian areas, as well as to the sub-occipital surface (38). Our study revealed that apart from the cortical branches, the caudal cerebral artery also issues small branches that extend to the middle cerebellar peduncle, the choroid plexus of the fourth verticle, and the brainstem. The multiple branches and anastomosis create plexiform patterns on the medullary surface. The observed variability in the origin and branching patterns of the terminal branches highlights the complexity of cerebellar vascularization in dromedary camels. While the overall arterial supply patterns were consistent, subtle inter-individual differences, particularly in forming the terminal branches, underscore the cerebellar arterial network's adaptability and potential evolutionary significance. These findings provide a foundation for further exploration of functional implications related to these variations.

The labyrinthine artery is a small branch from the rostral root of the caudal cerebellar artery. Kanan (19) and Salomon et al. (39) reported that these arteries supply the internal ear. This is a direct branch of the basilar artery in dogs (32). However, it is only a minor branch from the caudal cerebellar artery in dromedary camels.

In this study, the naming of the rostral and caudal cerebellar arteries and their branches in dromedary camels was aligned with the Nomina Anatomica Veterinaria (NAV) (40) to ensure consistency and facilitate comparative interpretation within the context of animal anatomy. Additionally, the Terminologia Anatomica (TA) (41) was used to compare our results with those of human literature.

While this study offers a detailed description of the cerebellar arteries in dromedary camels, it is essential to acknowledge a significant limitation. The lack of comprehensive studies describing the cerebellar arterial system in the camel and other animal species, particularly concerning the detailed branching patterns and nomenclature of the cerebellar arteries, restricted our ability to conduct in-depth comparative analyses and draw broader conclusions about evolutionary trends or interspecies variations.

In conclusion, the cerebellum of camels was supplied exclusively from the rostral and caudal cerebellar arteries, originating from the basilar artery. This study presented the first detailed map of the cerebellar arterial origins, courses, and branches in camels, thereby addressing a significant gap in the understanding of their neurovascular anatomy. These findings provide opportunities for future research on the functional implications of this unique vascular arrangement and support future research aimed at developing a detailed mapping of the cerebellar external architecture, similar to recent publications in the field (42). Moreover, this in-depth anatomical knowledge can be a basis for comparative studies across various animal species, potentially uncovering evolutionary adaptations and species-specific variations in cerebellar blood supply.

## Data Availability

The raw data supporting the conclusions of this article will be made available by the authors, without undue reservation.
